# Naringenin attenuates cerebral ischemia/reperfusion injury by inhibiting oxidative stress and inflammatory response via the activation of SIRT1/FOXO1 signaling pathway in vitro

**DOI:** 10.1590/acb380823

**Published:** 2023-05-01

**Authors:** Peng Zhao, Yi Lu, Zhiyun Wang

**Affiliations:** 1 Tianjin First Central Hospital – Department of Neurology – Tianjin, China.

**Keywords:** Oxidative Stress, Protective Agents, Brain Ischemia, Inflammation Mediators, Signal Transduction

## Abstract

**Purpose:**

To explore the protection of naringenin against oxygen-glucose deprivation/reperfusion (OGD/R)-induced HT22 cell injury, a cell model of cerebral ischemia/reperfusion (I/R) injury *in vitro*, focusing on SIRT1/FOXO1 signaling pathway.

**Methods:**

Cytotoxicity, apoptosis, reactive oxygen species (ROS) generation, malondialdehyde (MDA) content, 4-hydroxynonenoic acid (4-HNE) level, superoxide dismutase (SOD), glutathione peroxidase (GSH-Px) and catalase (CAT) activities were measured by commercial kits. Inflammatory cytokines levels were determined by enzyme-linked immunosorbent assay (ELISA). The protein expressions were monitored by Western blot analysis.

**Results:**

Naringenin significantly ameliorated OGD/R-induced cytotoxicity and apoptosis in HT22 cells. Meanwhile, naringenin promoted SIRT1 and FOXO1 protein expressions in OGD/R-subjected HT22 cells. In addition, naringenin attenuated OGD/R-induced cytotoxicity, apoptosis, oxidative stress (the increased ROS, MDA and 4-HNE levels, and the decreased SOD, GSH-Px and CAT activities) and inflammatory response (the increased tumor necrosis factor-α, interleukin [IL]-1β, and IL-6 levels and the decreased IL-10 level), which were blocked by the inhibition of the SIRT1/FOXO1 signaling pathway induced by SIRT1-siRNA transfection.

**Conclusions:**

Naringenin protected HT22 cells against OGD/R injury depending on its antioxidant and anti-inflammatory activities via promoting the SIRT1/FOXO1 signaling pathway.

## Introduction

Ischemic stroke is a common vascular disease resulting in death and long-term disability worldwide^1^^,^ 2. Thrombolytic therapy is known as the most effective therapeutic approach for ischemic stroke^3^. However, restoration of blood flow (reperfusion) unavoidably results in brain damage remains unavoidable, commonly known as cerebral ischemia-reperfusion (I/R) injury^4^^,^ 5. Although various pathological processes are participated in the progression of cerebral I/R injury, such as oxidative/nitrative stress, inflammation, necrosis, and apoptosis, to date, the molecular mechanisms of cerebral I/R injury are complex and remain to be fully understood^6^^,^ 7. In addition, due to the narrow therapeutic time window, few effective therapeutic strategies are available to prevent cerebral I/R injury^8^^,^ 9. Therefore, exploring novel safe and effective therapeutic medication for cerebral I/R injury and further demonstrating the underlying protective mechanisms are highly urgent and important.

Flavonoids are the major class of polyphenols and a broad range of experimental data have indicated the potential function of flavonoids in the management of cerebral I/R injury^10^^-^ 12. Naringenin (4,5,7-trihydroxy-flavanone) is one of the most critical naturally-occurring flavonoids widely present in natural products such as cherries, citrus fruits, and tomatoes^13^^-^ 15. Naringenin possesses various pharmacological properties, including antioxidant, anti-inflammatory, antiapoptotic, and neuroprotective properties^16^^-^ 18. Recently, Raza et al. also proved that naringenin abrogates brain injury in experimental stroke by suppressing NF-κB-mediated neuroinflammation^19^, indicating the potential neuroprotectant of naringenin in patients at high risk of ischemic stroke. However, the regarding therapeutic potential of naringenin in cerebral I/R injury is a critical limitation and the mechanisms underlying remain to be clarified.

Research report that silent information regulator 1 (SIRT1) is a class III histone deacetylase involved in regulating oxidative stress, apoptosis, inflammatory responses, and neuroprotective effect via modulating downstream target such as the forkhead box O1 (FOXO1)^20^^-^ 22. Recently, it has been proved that SIRT1/FOXO1 signaling pathway elicits neuroprotective function in many neurodegenerative disorders, including cerebral I/R injury^21^^,^ 23. SIRT1/FOXO1 signaling pathway was remarkably reduced after cerebral I/R injury, and promoting the SIRT1-FOXO1 signaling pathway contributes to the neuroprotective effect of electroacupuncture^24^ and calycosin-7-*O-*β*-D*-glucoside^21^ on cerebral I/R injury. Notably, it has been confirmed that a therapeutic approach with naringenin may have positive impacts on multiple diseases via targeting SIRT1^25^^-^ 27. Hence, the present further investigated whether naringenin attenuates cerebral I/R injury via activating SIRT1/FOXO1 signaling pathway.

Hence, the present study evaluated the neuroprotective effects of naringenin on hypoxia/reoxygenation (oxygen-glucose deprivation/reperfusion, OGD/R)-induced neurotoxicity in HT22 cells focusing on the SIRT1/FOXO1 signaling pathway. The results revealed that naringenin attenuated OGD/R injury and apoptosis by inhibiting oxidative stress and inflammatory response via the activation of SIRT1/FOXO1 signaling pathway in HT22 cells. These findings will give an insight into a promising potential therapeutic avenue of naringenin for cerebral I/R injury.

## Methods

### Cell culture

Mouse hippocampal neuronal cell line (HT22) was provided by American Type Culture Collection (ATCC, Manassas, VA, USA), and cultured in Dulbecco’s modified eagle medium (DMEM; Invitrogen, Carlsbad, CA, USA) supplemented with 10% fetal bovine serum (FBS; Invitrogen) and 100 U/mL streptomycin/penicillin (Gibco, Grand Island, NY, USA) in a humidified atmosphere at 37 °C in 5% CO_2_.

### OGD/R injury model

The cell model of cerebral I/R injury was established with HT22 cells which underwent OGD/R injury as described previously^28^. To initiate oxygen-glucose deprivation (OGD), HT22 cells were incubated in glucose-free DMEM and then placed in an anaerobic chamber (Heraeus, Hanau, Germany) for 6 h under an atmosphere of 95% N_2_/5% CO_2_ at 37 °C. After that, HT22 cells were restored with complete DMEM and recovered at normoxic conditions under 95% air/5% CO_2_ at 37 °C for reperfusion (24 h). The control groups were maintained in complete DMEM and no oxygen deprivation.

### siRNA interference

The specific siRNA targeting against SIRT1 (SASI_Hs01_00153666) and negative scramble siRNA (SIC001) designed and synthesized by Santa Cruz Biotechnology Co. (Santa Cruz, CA, USA). SiRNA transfection was carried out by Lipofectamine 2000 (Invitrogen, Carlsbad, CA, USA, 11668–027) according to the manufacturer’s instructions. After siRNA transfection for 48 h, the transfection efficiency was determined by western blotting analysis.

### Cell viability assays

The cell viability was measured by a Cell Counting Kit-8 (CCK-8) assay (Dojindo Laboratories, Kumamoto, Japan). Briefly, CCK-8 solution (10 μL) was added to each well and incubated at 37 °C for 2 h. Then, the absorbance at 450 nm was determined with an automatic microplate reader (PerkinElmer Victor 1420, Alburg, VT, United States). Cell viability was given as a percentage absorbance of untreated control cells.

### Lactate dehydrogenase (LDH) release assay

The release of LDH from HT22 cells was determined using the LDH Cytotoxicity Assay kit (Beyotime Biotechnology, Shanghai, China) by following the manufacturer’s protocol. The absorbance at 490 nm was measured using a microplate reader (Bio-Rad, Hercules, California, USA).

### Quantitative RT-PCR

The total RNA from HT22 cells was isolated using TRIzol Reagent (Invitrogen, 15596018). Then, total RNA (2 μg) was reverse transcribed into cDNA using the PrimeScript RT Reagent kit with gDNA Eraser (Takara) at 42 °C for 15 min and 85 °C for 5 min. Quantitative polymerase chain reaction (PCR) was performed using a PikoReal 96 Real-Time PCR system (Thermo Fisher Scientific, Inc.) with Power SYBR Green Master Mix (Applied Biosystems; Thermo Fisher Scientific, Inc.). The sequences of the primers were as follows: SIRT1 forward, 5’-CAGCTCTGCTACAATTCATCGCGTC-3’ and reverse, 5’-AATCTCTGTAGAGTCCAGCGCGTGTG-3’; GAPDH forward, *5’- GACCTGCCGTCTAGAAAAAC -3’* and reverse, *5’- CTGTAGCCAAATTCGTTGTC-3’.* Relative quantitative analysis in the mRNA expression was calculated using the 2^ΔΔCq^ method and normalized to GAPDH.

### Annexin V/PI flow cytometric analysis

The percentage of cells actively undergoing apoptosis was detected by the Annexin V-FITC/PT Apoptosis Detection Kit (Baosai Biotech, Shanghai, China) according to the manufacturer’s instructions. In brief, treated HT22 cells were collected, washed twice with phosphate buffered saline (PBS), and resuspended in binding buffer (100 μL). Then, Annexin V-FITC (5 μL) and PI (10 μL) were added to the cell suspension (1–5 × 10^6^·mL^–1^, 100 μL) and incubated for 20 min in darkness. Apoptosis rate was quantified by a FACSCanto II flow cytometry (Biomerry Biotechnology Co., Ltd., Beijing, China) and analyzed by FlowJo (version 7.6.1; FlowJo LLC) software.

### Measurement of reactive oxygen species (ROS) generation

Intracellular ROS generation was assessed by a ROS assay kit (Beyotime Institute of Biotechnology, Shanghai, China) with 2’,7’-dichlorodihydrofluorescein diacetate (DCFH-DA) staining, according to the manufacturer’s protocol. DCFH-DA is converted to DCFH by intracellular esterases, which is subsequently converted to the fluorescent 2’,7’-dichlorofluorescein (DCF) by ROS. Following drug treatment, HT22 cells (at a density of 2.5 × 10^5^ cells/well) were incubated in serum-free DMEM containing DCFH-DA (10 μmol·L^–1^) for 20 min at 37 °C in in the dark. After washing 3 times in PBS (5 min each), the fluorescent was analyzed by a fluorescence microscope (HCS, Thermo Fisher, scientific, Waltham, MA, USA). The relative fluorescence intensity was determined using a FACSCanto II flow cytometry (Biomerry Biotechnology Co., Ltd., Beijing, China).

### Quantification of malondialdehyde (MDA) content, 4-hydroxynonenoic acid (4-HNE) level, SOD, glutathione peroxidase (GSH-Px), and catalase (CAT) activities

HT22 cells were collected, homogenized in PBS, and homogenized with a homogenizer machine. After centrifugation at 3000 rpm for 15 min, the supernatant was collected to detect the levels of MDA and 4-HNE, and the activities of SOD and GSH-Px by MDA assay kit, 4-HNE, SOD assay kit, GSH-Px assay kit, and CAT assay kit (Nanjing Jiancheng Biotech, Nanjing, China) respectively, according to the manufacturer’s instructions.

### Enzyme-linked immunosorbent assay (ELISA)

According to the protocols (Nanjing Jiancheng Bioengineering Institute, Nanjing, China), specific ELISA kits were used to detect the level of proinflammatory cytokines TNF-α, IL-lβ, and IL-6 and anti-inflammatory factor IL-10 in the cell culture supernatant. The optical density was measured at 450 nm with an automatic microplate reader (PerkinElmer Victor 1420, Alburg, VT, United States) and the data were used to calculate the levels of various cytokines based on the standard curves.

### Western blotting analysis

HT22 cells were lysed with RIPA Lysis Buffer (Beyotime Biotechnology, Shanghai, China) for 30 min on ice and were centrifuged at 10,000 × g for 30 min at 4 °C. The supernatants were harvested, and then the protein content was determined with a bicinchoninic acid protein assay kit (Beyotime Biotechnology). The total protein (30 μg) was separated by SDS-PAGE and then transferred onto PVDF membranes (Millipore, Billerica, MA, United States). Membranes were blocked with 5% nonfat milk with PBS/0.1% Tween, followed by incubation overnight with anti-Bax (1:2,000; Cell Signaling Technology, Boston, MA, United States), anti-Bcl-2 (1:2,000; Cell Signaling Technology), anti-SIRT1 (1:1000; Abcam, Cambridge, United Kingdom), anti-FOXO1 (1:1000; Abcam), and anti-GAPDH (1:5,000; Cell Signaling Technology) antibodies at 4 °C. After washing with PBS/0.1% Tween, the membranes were incubated with horseradish peroxidase-labeled secondary antibody (1:2,000; Cell Signaling Technology) for 2 h at room temperature. After washing with PBS/0.1% Tween again, the imaging was performed with the application of Enhanced chemiluminescence substrate (ECL substrate; Beyotime Biotechnology). The gray density of protein bands was analyzed by Image J (National Institutes of Health, Bethesda, MD, United States) and then normalized to the GAPDH loading control.

### Statistical analysis

All experimental data are presented as mean ± standard deviation (SD) from at least three independent. Statistical analyses were performed by SPSS software version 22.0 (SPSS, Inc. IBM Corporation). The normal distribution and variance homogeneity were measured. Statistical differences between the two groups were analyzed by one-way analysis of variance followed by Tukey’s test. For nonnormally distributed data and/or non-homogeneous variance, Kruskal-Wallis test was used. P < 0.05 was regarded as statistically significant.

## Results

### Naringenin attenuates OGD/R-induced HT22 cells injuries

**Figure 1:**
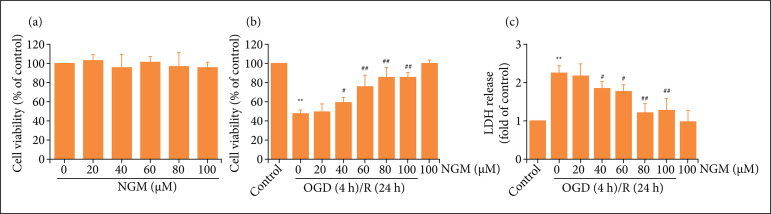
Effects of naringenin on cell viability and LDH release in OGD/R-exposed HT22 cells. HT22 cells were treated with different concentrations of naringenin (20, 40, 60, 80, or 100 μmol·L^–1^) for 24 h, and **(a)** cell viability was detected by CCK-8 assay. HT22 cells were treated with naringenin (20, 40, 60, 80 or 100 μmol·L^–1^) for 2 h followed by treatment with OGD (6 h)/R (24 h); **(b)** cell viability was detected by CCK-8 assay; and **(c)** LDH releases was assayed by LDH cytotoxicity assay kit. Data were expressed as mean ± SD from 3–4 independent experiments. ^**^P < 0.01 vs. control group; ^#^ P< 0.05, ^##^P < 0.01 vs. OGD/R group. NGN: naringenin; OGD/R: oxygen-glucose deprivation/reperfusion.

To investigate the protection of naringenin against OGD/R injury, the effect of different concentrations of naringenin (20, 40, 60, 80, or 100 μmol·L^–1^) on cell viability was first investigated. Results showed that compared with the control group, naringenin (20, 40, 60, or 80 μmol·L^–1^) alone treatment did not affect cell viability (Fig. 1a). Then, results further found that naringenin (40, 60, 80, and 100 μmol·L^–1^) pretreatment reversed OGD/R-induced the down-regulation of cell viability (Fig. 1b) and the up-regulation of LDH releases (Fig. 1c) in HT22 cells. Naringenin (80 μmol·L^–1^) significantly increased HT22 cell viability and decreased LDH release under OGD/R (P < 0.01), which was used for the subsequent experiments. These results indicated that naringenin protects HT22 cells against OGD/R injury.

### Naringenin inhibits OGD/R-induced apoptosis in HT22 cells

**Figure 2:**
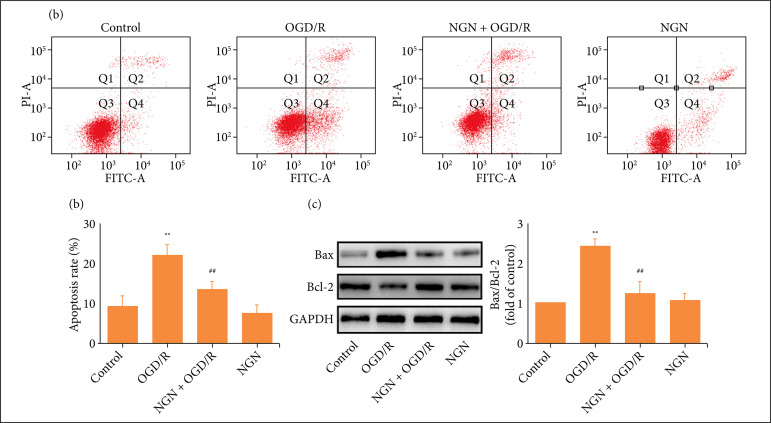
Effects of naringenin on OGD/R-induced apoptosis in HT22 cells. HT22 cells were treated with naringenin (80 μmol·L^–1^) for 2 h followed by treatment with OGD (6 h)/R (24 h). **(a)** The apoptosis rate was detected by Annexin V-FITC/PI double staining followed by flow cytometry; **(b)** Quantitative analysis of early apoptosis (Annexin V-FITC^+^/PI^-^) and late apoptosis (Annexin V-FITC^+^/PI^+^); **(c)** Protein expression was analyzed by western blot, and GAPDH was used as an internal control. Data were expressed as mean ± SD from 3-4 independent experiments. ^**^P < 0.01 vs. control group; ^##^P < 0.01 vs. OGD/R group. NGN: naringenin; OGD/R: oxygen-glucose deprivation/reperfusion.

Next, we further demonstrated the effect of naringenin on apoptosis in OGD/R-treated HT22 cells. Annexin V-FITC/PI double staining results revealed that pretreatment with naringenin significantly blocks OGD/R-induced an increase in apoptosis rate (Fig. 2a). In addition, western blot analysis results found that naringenin also reversed OGD/R-resulted in the up-regulation of pro-apoptotic protein Bax expression (Fig. 2b) and the down-regulation of anti-apoptotic proteins Bcl-2 expression (Fig. 2c) in HT22 cells. These data suggested that naringenin blocks OGD/R-induced apoptosis in HT22 cells.

### Naringenin promotes SIRT1/FOXO1 signaling pathway in OGD/R-exposed HT22 cells

**Figure 3:**
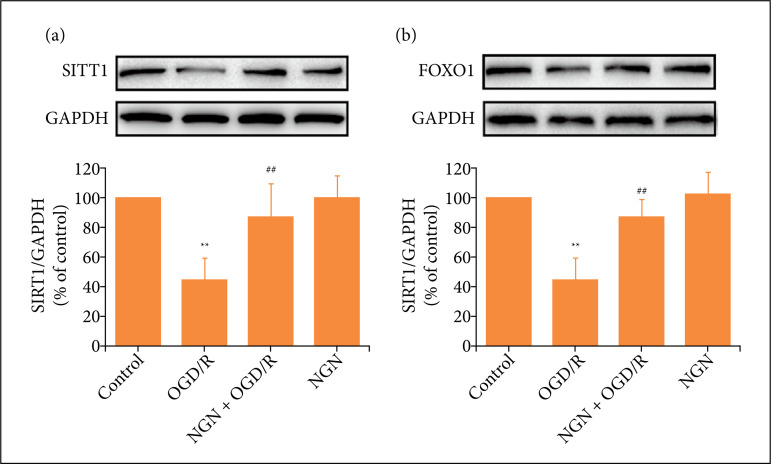
Effects of naringenin on SIRT1/FOXO1 signaling pathway in OGD/R-exposed HT22 cells. HT22 cells were treated with naringenin (80 μmol·L^–1^) for 2 h followed by treatment with OGD (6 h)/R (24 h). **(A)** SIRT1 and **(B)** FOXO1 expressions were detected by western blot analysis. Data were expressed as mean ± SD from 3-4 independent experiments. ^**^P < 0.01 vs. control group; ^##^P < 0.01 vs. OGD/R group. NGN: naringenin; OGD/R: oxygen-glucose deprivation/reperfusion.

Silent information regulator 1 (SIRT1) has been shown to play a significant role in neuroprotection against cerebral I/R injury via regulating FOXO1^21^^,^ 24. To explore the underlying mechanisms by which pretreatment with naringenin may inhibit OGD/R injury via the SIRT1/FOXO1 signaling pathway, the protein expressions of SIRT1 and FOXO1 were measured. Results showed that OGD/R remarkably reduced the expression levels of SIRT1 (Fig. 3a) and FOXO1 (Fig. 3a) in HT22 cells compared with the control group. However, naringenin pretreatment obviously increased the expression levels of SIRT1 (Fig. 3a) and FOXO1 (Fig. 3b) compared with the OGD/R group. Naringenin alone did not affect the expression levels of SIRT1 and FOXO1 compared with the control group. These results implied that naringenin promotes SIRT1/FOXO1 signaling pathway in OGD/R-exposed HT22 cells.

### Inhibition of SIRT1/FOXO1 signaling pathway restricted the protective effect of naringenin on OGD/R injury in HT22 cells

**Figure 4:**
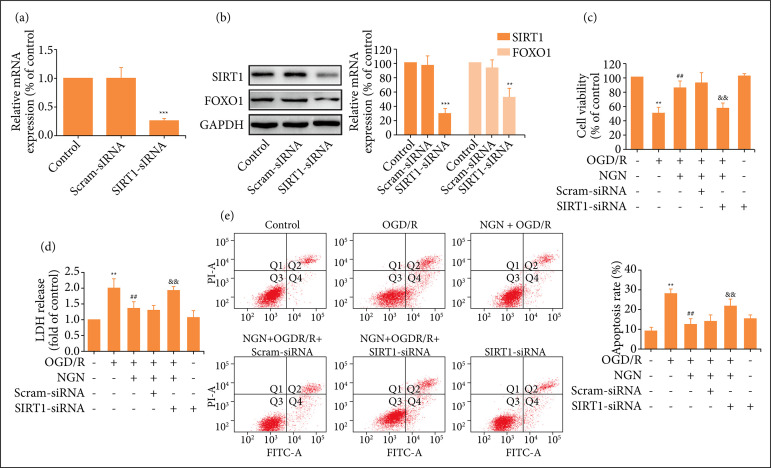
Effects of SIRT1-siRNA transfection on the protection of naringenin against OGD/R injury in HT22 cells. After transfection with SIRT1-siRNA (50 nmol·L^–1^) or scrambled siRNA (Scram-siRNA; 50 nmol·L^–1^) for 48 h, **(a)** the level of SRT1 mRNA was measured by Quantitative RT-PCR and **(b)** the expressions of SIRT1 and FOXO1 proteins were detected by western blot analysis. Data were expressed as mean ± SD from 3-4 independent experiments. ^**^P < 0.01, ^***^P < 0.001 vs. Scram-siRNA group. HT22 cells were transfected with SIRT1-siRNA (50 nmol·L^–1^) or Scram-siRNA (50 nmol·L^–1^) for 6 h and then treated with naringenin (80 μmol·L^–1^) for 2 h followed by treatment with OGD (6 h)/R (24 h); **(c)** Cell viability was detected by CCK-8 assay and **(d)** LDH releases were assayed by LDH cytotoxicity assay kit; **(e)** The apoptosis rate was detected by Annexin V-FITC/PI double staining followed by flow cytometry. Data were expressed as mean ± SD from 3-4 independent experiments. ^**^P < 0.01 vs. control group; ^##^P < 0.01 vs. OGD/R group; ^&&^P < 0.01 vs. NGN + OGD/R + Scram-siRNA group. NGN: naringenin; OGD/R: oxygen-glucose deprivation/reperfusion.

To further confirm the role of the SIRT1/FOXO1 signaling pathway in the neuroprotection of naringenin against OGD/R injury, HT22 cells were transfected with siRNAs targeting SIRT1 (SIRT1-siRNA). Results revealed that compared with the scrambled control siRNA (Scram-siRNA), SIRT1-siRNA transfection remarkably reduces the level of SIRT1 mRNA (Fig. 4a). In addition, SIRT1-siRNA transfection also significantly attenuated the expressions of SIRT1 and FOXO1 protein compared with Scram-siRNA transfection (Fig. 4b), indicating SIRT1-siRNA successfully suppressed SIRT1/FOXO1 signaling pathway in HT22 cells. On this basis, results further found that SIRT1-siRNA transfection significantly reverses naringenin-induced an increase in cell viability (Fig. 4c) and LDH release (Fig. 4d) compared with scrambled siRNA transfection. Next, the role of the SIRT1/FOXO1 signaling pathway in the anti-apoptotic function of naringenin in OGD/R injury was explored. As shown in Fig. 4e, knockdown of SIRT1/FOXO1 signaling pathway induced by SIRT1-siRNA transfection attenuates naringenin-decreased the apoptosis rate compared to scrambled siRNA transfection in OGD/R-subjected HT22 cells. All in all, these results indicated that SIRT1/FOXO1 signaling pathway mediates the protection of naringenin against OGD/R injury in HT22 cells.

### Blockage of SIRT1/FOXO1 signaling pathway mitigates the inhibition of naringenin on OGD/R-induced oxidative stress in HT22 cells

**Figure 5:**
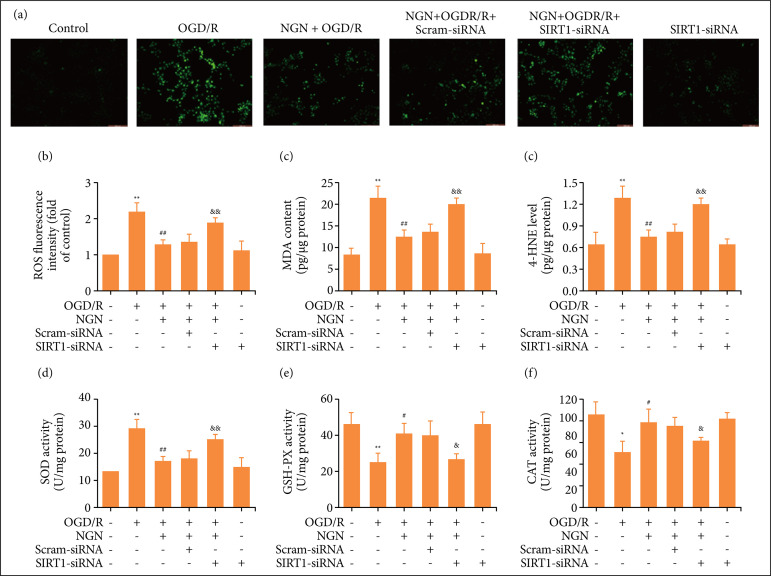
Effects of SIRT1-siRNA transfection on the inhibition of naringenin on oxidative stress in OGD/R-subjected HT22 cells. After transfection with SIRT1-siRNA(50 nmol·L^–1^) or scrambled siRNA (50 nmol·L^–1^) for 6 h, HT22 cells were treated with naringenin (80 μmol·L^–1^) for 2 h followed by treatment with OGD (6 h)/R (24 h). **(a)** ROS generation was calculated by DCFH-DA staining and **(b)** quantitative analysis of ROS fluorescence by flow cytometry; **(c)** MDA content; **(d)** 4-HNE level; **(e)** SOD activity; **(f)** GSH-Px activity, and CAT activity was determined by MDA assay kit, 4-hydroxynonenal ELISA kit, SOD assay kit, glutathione peroxidase (GSH-Px) assay kit, and CAT assay kit, respectively. Data were expressed as mean ± SD from three independent experiments. ^*^P < 0.05, ^**^P < 0.01 vs. control group; ^#^P < 0.05, ^##^P < 0.01 vs. OGD/R group; ^&^P < 0.05, ^&&^P < 0.01 vs. NGN + OGD/R +Scram-siRNA group. NGN: naringenin; OGD/R: oxygen-glucose deprivation/reperfusion.

It has been confirmed that oxidative stress plays a significant role in brain damage after stroke^29^. Then, we investigated the effects of naringenin on oxidative stress in OGD/R-exposed HT22 cells and the role of the SIRT1/FOXO1 signaling pathway in this process. Results found that naringenin significantly decreased endogenous ROS generation in OGD/R-treated HT22 cells, while inhibition of SIRT1/FOXO1 signaling pathway induced by SIRT1-siRNA transfection increased endogenous ROS production in HT22 cells cotreated naringenin and OGD/R group (Fig. 5a, b). MDA and 4-HNE, two lipid peroxidations, reflect ROS-dependent tissue damage^30^. Naringenin also attenuated OGD/R-induced increases of MDA (Fig. 5c) and 4-HNE (Fig. 5d) levels, which were blocked by SIRT1-siRNA transfection compared with scrambled siRNA transfection. Furthermore, to confirm the protection of naringenin against oxidative stress under OGD/R condition, the effects of naringenin on the activities of antioxidant enzymes including SOD, GSH-PX, and CAT were explored. Results revealed that naringenin mitigated OGD/R-induced decreases in the activities of SOD (Fig. 5e), GSH-PX (Fig. 5f), and CAT (Fig. 5g) in HT22 cells. However, these functions of naringenin were also reversed by SIRT1-siRNA transfection. SIRT1-siRNA transfection alone had no effect on oxidative stress compared with the control group. All in all, these results indicated that naringenin attenuates OGD/R-induced oxidative stress via promoting the SIRT1/FOXO1 signaling pathway in HT22 cells.

### Blockage of SIRT1 signaling pathway reverses the inhibition of naringenin on OGD/R-induced inflammatory response in HT22 cells

**Figure 6:**
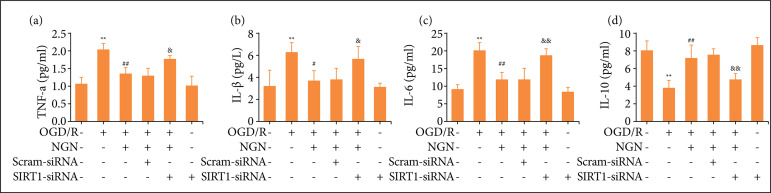
Effects of SIRT1-siRNA transfection on the inhibition of naringenin on inflammatory response in OGD/R-treated HT22 cells. After transfection with SIRT1-siRNA (50 nmol·L^–1^) or scramble siRNA (50 nmol·L^–1^) for 6 h, HT22 cells were treated with naringenin (80 μmol·L^–1^) for 2 h followed by treatment with OGD (6 h)/R (24 h). The levels of inflammation-related factors including **(a)** TNF-α, **(b)** IL-lβ, **(c)** IL-6 and **(d)** IL-10 were measured by enzyme-linked immunosorbent assay (ELISA). Data were expressed as mean ± SD from three independent experiments. ^**^P < 0.01 vs. control group; ^#^P < 0.05, ^##^P < 0.01 vs. OGD/R group; ^&^P < 0.05, ^&&^P < 0.01 vs. NGN + OGD/R + Scram-siRNA group. NGN: naringenin; OGD/R: oxygen-glucose deprivation/reperfusion.

Neuroinflammation plays a very important role in the pathogenesis of cerebral I/R injury^31^. To explore the potential anti-inflammatory effects of naringenin in OGD/R-treated HT22 cells and the role of SIRT1 signaling in this process, the levels of pro-inflammatory cytokines, including TNF-α, IL-1β and IL-6, and the anti-inflammatory cytokine, including IL-10 were measured. As shown in Fig. 6, naringenin pretreatment significantly reduced the levels of TNF-α (Fig. 6a), IL-1β (Fig. 6b), and IL-6 (Fig. 6c) compared with OGD/R group, while SIRT1-siRNA transfection obviously increased the levels of TNF-α (Fig. 6a), IL-1β (Fig. 6b) and IL-6 (Fig. 6c) compared with HT22 cells cotreated with naringenin and OGD/R plus scrambled siRNA transfection. In addition, SIRT1-siRNA transfection also reversed naringenin-induced the down-regulated level of IL-10 (Fig. 6d) in OGD/R-treated HT22 cells compared with scrambled siRNA transfection. SIRT1-siRNA transfection alone had no effect on inflammatory response compared with the control group. These results suggested that naringenin attenuates OGD/R-induced inflammatory response via promoting the SIRT1/FOXO1 signaling pathway in HT22 cells.

## Discussion

The present study demonstrated that naringenin inhibits OGD/R-induced oxidative stress and inflammation, rescuing neuronal cell death. The mechanism was involved the promoting of the SIRT1/FOXO1 signaling pathway. The results of this pilot research underline the potential of naringenin in treating cerebral I/R injury.

Naringenin is a natural flavonoid contained widely present in citrus fruit and grapefruits. Increasing evidence reveals the neuroprotective function of naringenin *in vivo* and *in vitro* studies on several neurological disorders^32^^,^ 33. Naringenin attenuates lipopolysaccharide-induced dopamine neurotoxicity in Parkinson’s disease^34^, β-amyloid-induced neurotoxicity in Alzheimer’s disease^35^, and experimental ischemic stroke^36^. However, the protection of naringenin against cerebral I/R injury remains unclear. In the present study, the result first revealed that naringenin pretreatment mitigates OGD/R-induced cytotoxicity and apoptosis in HT22 cells. Brain ischemia leads to the up-regulation of the proapoptotic protein Bax expression and down-regulation of anti-apoptotic protein expression, then activating apoptotic cell death^37^. In addition, the present results found that naringenin decreases Bax expression and increases Bcl-2 expression in OGD/R-exposed HT22 cells. These results indicated that naringenin attenuates cerebral I/R injury via inhibiting apoptosis *in vitro*.

Several studies have pointed out that SIRT1 plays a neuroprotective effect on I/R injury, and their expression in the brain is reduced exposed to multiple stress stimuli that collectively drive the development of brain ischemia^38^^-^ 40. Similarly, the present study showed that the expression of SIRT1 was also down-regulated in OGD/R-treated HT22 cells and recovered after naringenin treatment, which is consistent with previous study that naringenin promotes SIRT1 expression^27^. As the first found transcription factor in the FOXO family, FOXO1 was implicated as a crucial regulator of neuron survival in brain ischemia and is also a downstream target of SIRT1^41^. Our result also showed that OGD/R results in the down-regulation of FOXO1 expression, which is also reserved by naringenin. Recently, more and more studies have confirmed that activating the SIRT1/FOXO1 signaling pathway contributes to protecting against cerebral I/R injury^21^^,^ 24. In the present study, the results further found that SIRT1-siRNA transfection leads to the inhibition of the SIRT1/FOXO1 signaling pathway, and then blocks naringenin-elicited protection against OGD/R injury and apoptosis. Taken together, these discoveries impacted that SIRT1/FOXO1 signaling pathway mediates the protection of naringenin against OGD/R injury in HT22 cells.

For decades, numerous clinical trials have evaluated that oxidative stress plays crucial roles in the occurrence and development of cerebral I/R injury and the impairment of antioxidant defense system including SOD, GSH-Px, and CAT activities endogenously formed by I/R are most applicable^42^^-^ 44. Naringenin is neuroprotective against a variety of neurological diseases associated with antioxidant mechanisms and inhibition of lipid peroxidation^45^^-^ 47. This study also revealed that naringenin pretreatment reverses OGD/R-induced increases in ROS generation and lipid peroxidation (MDA and 4-HNE levels) and the decreases in SOD, GSH-Px, and CAT activities in HT22 cells, indicating the antioxidative function of naringenin in cerebral I/R injury. Besides, a prevision study reveals that the enhanced SIRT1/FOXO1 signaling pathway can play a role in exerting anti-oxidative stress and protecting cerebral ischemia injury^21^. However, the role of the SIRT1/FOXO1 signaling pathway in the anti-oxidative activity of naringenin during cerebral I/R injury has not been explored. In the present study, the results further found that inhibition of the SIRT1/FOXO1 signaling pathway blocks naringenin-induced inhibition of oxidative stress and the promotion of anti-oxidant activity in OGD/R-treated HT22 cells. All in all, these results suggested that naringenin protects HT22 cells against OGD/R injury via reducing oxidative stress via enhancing the SIRT1/FOXO1 signaling pathway.

It has been reported that along with oxidative damage, inflammation is important in the pathophysiological variations that take place after cerebral I/R injury and provoking the death of the nerve cells^31^^,^ 48. Cerebral ischemia produces great amounts of proinflammatory factors such as TNF-α and IL-1β and inhibits the production of anti-inflammatory factors such as IL-10 and IL-4^49^. Although the anti-inflammatory activity of naringenin has been confirmed^18^^,^ 50, the inhibition of naringenin on OGD/R-induced inflammatory response has not been reported. The present study revealed that naringenin pretreatment also attenuates OGD/R-induced increases in TNF-α, IL-1β, and IL-6 levels, and a decrease in IL-10 level. However, these actions of naringenin were mitigated by inhibition of the SIRT1/FOXO1 signaling pathway induced by SIRT1-siRNA transfection, which was consistent with previous studies that activating the SRT1/FOXO1 signaling pathway mediates the beneficent effects of other flavonoids^51^^,^ 52. Taken together, these results indicated that naringenin protects HT22 cells against OGD/R-induced inflammatory injury via promoting the SIRT1/FOXO1 signaling pathway.

## Conclusion

All in all, the present experiments successfully elucidated the mechanism of action by which naringenin attenuates oxidative stress and inflammatory response, and then protects against cerebral I/R injury *in vitro*, namely, by enhancing the SIRT1/FOXO1 signaling pathway. These data provide insight into the role of naringenin in oxidative and inflammatory regulation in cerebral I/R-induced brain injury in the promotion of SIRT1/FOXO1 signaling pathway and promises the potential therapeutic candidate of naringenin in the treatment of brain injury associated with ischemic stroke.

## Data Availability

All dataset were generated or analyzed in the current study.

## References

[1] Lindsay MP, Norrving B, Sacco RL, Brainin M, Hacke W, Martins S, Pandian J, Feigin V. (2019). World Stroke Organization (WSO): Global Stroke Fact Sheet 2019. Int J Stroke.

[2] Donnan GA, Fisher M, Macleod M, Davis SM. (2008). Stroke. Lancet.

[3] Schregel K, Behme D, Tsogkas I, Knauth M, Maier I, Karch A, Mikolajczyk R, Bahr M, Schaper J, Hinz J, Liman J, Psychogios MN (2018). Optimized management of endovascular treatment for acute ischemic stroke. J Vis Exp.

[4] Wu MY, Yiang GT, Liao WT, Tsai AP, Cheng YL, Cheng PW, Li CY, Li CJ (2018). Current mechanistic concepts in ischemia and reperfusion injury. Cell Physiol Biochem.

[5] Hao Y, Xin M, Feng L, Wang X, Wang X, Ma D, Feng J. (2020). Review cerebral ischemic tolerance and preconditioning: Methods, mechanisms, clinical applications, and challenges. Front Neurol.

[6] Liu H, Wu X, Luo J, Zhao L, Li X, Guo H, Bai H, Cui W, Guo W, Feng D, Qu Y. (2020). Adiponectin peptide alleviates oxidative stress and NLRP3 inflammasome activation after cerebral ischemia-reperfusion injury by regulating AMPK/GSK-3beta. Exp Neurol.

[7] Galkin A. (2019). Brain ischemia/reperfusion injury and mitochondrial complex I damage. Biochemistry (Mosc).

[8] Hentia C, Rizzato A, Camporesi E, Yang Z, Muntean DM, Sandesc D, Bosco G. (2018). An overview of protective strategies against ischemia/reperfusion injury: The role of hyperbaric oxygen preconditioning. Brain Behav.

[9] Sun K, Fan J, Han J. (2015). Ameliorating effects of traditional Chinese medicine preparation, Chinese materia medica and active compounds on ischemia/reperfusion-induced cerebral microcirculatory disturbances and neuron damage. Acta Pharm Sin B.

[10] Ling C, Lei C, Zou M, Cai X, Xiang Y, Xie Y, Li X, Huang D, Wang Y (2020). Neuroprotective effect of apigenin against cerebral ischemia/reperfusion injury. J Int Med Res.

[11] Wang YY, Chang CY, Lin SY, Wang JD, Wu CC, Chen WY, Kuan YH, Liao SL, Wang WY, Chen CJ (2020). Quercetin protects against cerebral ischemia/reperfusion and oxygen glucose deprivation/reoxygenation neurotoxicity. J Nutr Biochem.

[12] Li Y, Wang R, Xue L, Yang Y, Zhi F. (2020). Astilbin protects against cerebral ischaemia/reperfusion injury by inhibiting cellular apoptosis and ROS-NLRP3 inflammasome axis activation. Int Immunopharmacol.

[13] Memariani Z, Abbas SQ, Ul Hassan, Ahmadi A, Chabra A (2020). Naringin and naringenin as anticancer agents and adjuvants in cancer combination therapy: Efficacy and molecular mechanisms of action, a comprehensive narrative review. Pharmacol Res.

[14] Gandhi GR, Vasconcelos ABS, Wu DT, Li HB, Antony PJ, Li H, Geng F, Gurgel RQ, Narain N, Gan RY (2020). Citrus Flavonoids as promising phytochemicals targeting diabetes and related complications: A systematic review of in vitro and in vivo studies. Nutrients.

[15] Heidary R, Samimi Z, Moradi SZ, Little PJ, Xu S, Farzaei MH (2020). Naringenin and naringin in cardiovascular disease prevention: A preclinical review. Eur J Pharmacol.

[16] Niu X, Sang H, Wang J. (2021). Naringenin attenuates experimental autoimmune encephalomyelitis by protecting the intact of blood-brain barrier and controlling inflammatory cell migration. J Nutr Biochem.

[17] Kesh S, Kannan RR, Balakrishnan A. (2021). Naringenin alleviates 6-hydroxydopamine induced Parkinsonism in SHSY5Y cells and zebrafish model. Comp Biochem Physiol C Toxicol Pharmacol.

[18] Rehman K, Khan II, Akash MSH, Jabeen K, Haider K. (2020). Naringenin downregulates inflammation-mediated nitric oxide overproduction and potentiates endogenous antioxidant status during hyperglycemia. J Food Biochem.

[19] Raza SS, Khan MM, Ahmad A, Ashafaq M, Islam F, Wagner AP, Safhi MM, Islam F (2013). Neuroprotective effect of naringenin is mediated through suppression of NF-κB signaling pathway in experimental stroke. Neuroscience.

[20] Singh V, Ubaid S. (2020). Role of silent information regulator 1 (SIRT1) in regulating oxidative stress and inflammation. Inflammation.

[21] Yan X, Yu A, Zheng H, Wang S, He Y, Wang L. (2019). Calycosin-7-*O-*β*-D*-glucoside attenuates OGD/R-induced damage by preventing oxidative stress and neuronal apoptosis via the SIRT1/FOXO1/PGC-1α pathway in HT22 cells. Neural Plast.

[22] Yang Y, Duan W, Li Y, Yan J, Yi W, Liang Z, Wang N, Yi D, Jin Z. (2013). New role of silent information regulator 1 in cerebral ischemia. Neurobiol Aging.

[23] Wang KJ, Zhang WQ, Liu JJ, Cui Y, Cui JZ (2020). Piceatannol protects against cerebral ischemia/reperfusion-induced apoptosis and oxidative stress via the Sirt1/FoxO1 signaling pathway. Mol Med Rep.

[24] Mei ZG, Huang YG, Feng ZT, Luo YN, Yang SB, Du LP, Jiang K, Liu XL, Fu XY, Deng YH, Zhou HJ (2020). Electroacupuncture ameliorates cerebral ischemia/reperfusion injury by suppressing autophagy *via* the SIRT1-FOXO1 signaling pathway. Aging (Albany NY).

[25] Miler M, Zivanovic J, Ajdzanovic V, Milenkovic D, Jaric I, Sosic-Jurjevic B, Milosevic V. (2020). Citrus flavanones upregulate thyrotroph sirt1 and differently affect thyroid Nrf2 expressions in old-aged Wistar Rats. J Agric Food Chem.

[26] Testai L, Piragine E, Piano I, Flori L, Da Pozzo E, Miragliotta V, Pirone A, Citi V, Di Cesare, Mannelli L, Brogi S, Carpi S, Martelli A, Nieri P, Martini C, Ghelardini C, Calderone V. (2020). The citrus flavonoid naringenin protects the myocardium from ageing-dependent dysfunction: Potential role of SIRT1. Oxid Med Cell Longev.

[27] Sarubbo F, Ramis MR, Kienzer C, Aparicio S, Esteban S, Miralles A, Moranta D. (2018). Chronic silymarin, quercetin and naringenin treatments increase monoamines synthesis and hippocampal Sirt1 levels improving cognition in aged rats. J Neuroimmune Pharmacol.

[28] Zhang S, Sun WC, Liang ZD, Yin XR, Ji ZR, Chen XH, Wei MJ, Pei L. (2020). LncRNA SNHG4 attenuates inflammatory responses by sponging miR-449c-5p and up-regulating STAT6 in microglial during cerebral ischemia-reperfusion injury. Drug Des Devel Ther.

[29] Chen H, Yoshioka H, Kim GS, Jung JE, Okami N, Sakata H, Maier CM, Narasimhan P, Goeders CE, Chan PH (2011). Oxidative stress in ischemic brain damage: mechanisms of cell death and potential molecular targets for neuroprotection. Antioxid Redox Signal.

[30] Michel F, Bonnefont-Rousselot D, Mas E, Drai J, Therond P. (2008). [Biomarkers of lipid peroxidation: analytical aspects]. Ann Biol Clin (Paris).

[31] Yang Q, Hu Z, Tang X (2019). Potential neuroprotective treatment of stroke: Targeting excitotoxicity, oxidative stress, and inflammation. Front Neurosci.

[32] Arafah A, Rehman MU, Mir TM, Wali AF, Ali R, Qamar W, Khan R, Ahmad A, Aga SS, Alqahtani S, Almatroudi NM (2020). Multi-therapeutic potential of naringenin (4’,5,7-trihydroxyflavonone): experimental evidence and mechanisms. Plants (Basel).

[33] Nouri Z, Fakhri S, El-Senduny FF, Sanadgol N, Abd-ElGhani GE, Farzaei MH, Chen JT (2019). On the neuroprotective effects of naringenin: Pharmacological targets, signaling pathways, molecular mechanisms, and clinical perspective. Biomolecules.

[34] Chen C, Wei YZ, He XM, Li DD, Wang GQ, Li JJ, Zhang F (2019). Naringenin produces neuroprotection against LPS-induced dopamine neurotoxicity via the inhibition of microglial NLRP3 inflammasome activation. Front Immunol.

[35] Md S, Gan SY, Haw YH, Ho CL, Wong S, Choudhury H. (2018). In vitro neuroprotective effects of naringenin nanoemulsion against β-amyloid toxicity through the regulation of amyloidogenesis and tau phosphorylation. Int J Biol Macromol.

[36] Bai X, Zhang X, Chen L, Zhang J, Zhang L, Zhao X, Zhao T, Zhao Y. (2014). Protective effect of naringenin in experimental ischemic stroke: down-regulated NOD2, RIP2, NF-kappaB, MMP-9 and up-regulated claudin-5 expression. Neurochem Res.

[37] Terashi T, Otsuka S, Takada S, Nakanishi K, Ueda K, Sumizono M, Kikuchi K, Sakakima H. (2019). Neuroprotective effects of different frequency preconditioning exercise on neuronal apoptosis after focal brain ischemia in rats. Neurol Res.

[38] Xu J, Jackson CW, Khoury N, Escobar I, Perez-Pinzon MA (2018). Brain SIRT1 mediates metabolic homeostasis and neuroprotection. Front Endocrinol (Lausanne).

[39] Meng X, Tan J, Li M, Song S, Miao Y, Zhang Q. (2017). Sirt1: Role under the condition of ischemia/hypoxia. Cell Mol Neurobiol.

[40] Koronowski KB, Perez-Pinzon MA (2015). Sirt1 in cerebral ischemia. Brain Circ.

[41] Al-Massadi O, Quinones M, Clasadonte J, Hernandez-Bautista R, Romero-Pico A, Folgueira C, Morgan DA, Kallo I, Heras V, Senra A, Funderburk SC, Krashes MJ, Souto Y, Fidalgo M, Luquet S, Chee MJ, Imbernon M, Beiroa D, Garcia-Caballero L, Gallego R, Lam BYH, Yeo G, Lopez M, Liposits Z, Rahmouni K, Prevot V, Dieguez C, Nogueiras R. (2019). MCH regulates SIRT1/FoxO1 and reduces POMC neuronal activity to induce hyperphagia, adiposity, and glucose intolerance. Diabetes.

[42] Li R, Rhee SJ, Bae S, Su S, Kang CS, Ke Q, Koo YE, Ryu C, Song CG, Lee D, Kang PM (2021). H_2_O_2_-responsive antioxidant nanoparticle attenuates whole body ischemia/reperfusion-induced multi-organ damages. J Cardiovasc Pharmacol Ther.

[43] Chen H, He Y, Chen S, Qi S, Shen J. (2020). Therapeutic targets of oxidative/nitrosative stress and neuroinflammation in ischemic stroke: Applications for natural product efficacy with omics and systemic biology. Pharmacol Res.

[44] Chen CH, Hsieh CL (2020). Effect of acupuncture on oxidative stress induced by cerebral ischemia-reperfusion injury. Antioxidants (Basel).

[45] Habtemariam S. (2019). The Nrf2/HO-1 axis as targets for flavanones: Neuroprotection by pinocembrin, naringenin, and eriodictyol. Oxid Med Cell Longev.

[46] Md S, Alhakamy NA, Aldawsari HM, Asfour HZ (2019). Neuroprotective and antioxidant effect of naringenin-loaded nanoparticles for nose-to-brain delivery. Brain Sci.

[47] Sugumar M, Sevanan M, Sekar S. (2019). Neuroprotective effect of naringenin against MPTP-induced oxidative stress. Int J Neurosci.

[48] Mo Y, Sun YY, Liu KY (2020). Autophagy and inflammation in ischemic stroke. Neural Regen Res.

[49] Chen S, Chen H, Du Q, Shen J. (2020). Targeting myeloperoxidase (MPO) mediated oxidative stress and inflammation for reducing brain ischemia injury: Potential application of natural compounds. Front Physiol.

[50] Bansal Y, Singh R, Saroj P, Sodhi RK, Kuhad A. (2018). Naringenin protects against oxido-inflammatory aberrations and altered tryptophan metabolism in olfactory bulbectomized-mice model of depression. Toxicol Appl Pharmacol.

[51] Chu Q, Yu X, Jia R, Wang Y, Zhang Y, Zhang S, Liu Y, Li Y, Chen W, Ye X, Zheng X. (2019). Flavonoids from *Apios americana* Medikus Leaves Protect RAW264.7 Cells against Inflammation via Inhibition of MAPKs, Akt-mTOR Pathways, and Nfr2 Activation. Oxid Med Cell Longev.

[52] Xu J, Chen Y, Xing Y, Ye S. (2019). Metformin inhibits high glucose-induced mesangial cell proliferation, inflammation and ECM expression through the SIRT1-FOXO1-autophagy axis. Clin Exp Pharmacol Physiol.

